# Effect of Layer and Film Thickness and Temperature on the Mechanical Property of Micro- and Nano-Layered PC/PMMA Films Subjected to Thermal Aging

**DOI:** 10.3390/ma8052062

**Published:** 2015-04-23

**Authors:** Ahmed Abdel-Mohti, Alison N. Garbash, Saad Almagahwi, Hui Shen

**Affiliations:** 1 Civil Engineering Department, Ohio Northern University, Ada, OH 45810, USA; E-Mail: a-abdel-mohti@onu.edu; 2 Mechanical Engineering Department, Ohio Northern University, Ada, OH 45810, USA; E-Mails: agarbash@gmail.com (A.N.G.); s-almagahwi@onu.edu (S.A.)

**Keywords:** multilayered polymer film, thermal aging, film thickness, layer thickness, aging temperature, mechanical properties

## Abstract

Multilayered polymer films with biomimicking, layered structures have unique microstructures and many potential applications. However, a major limitation of polymer films is the deterioration of mechanical properties in working environments. To facilitate the design and development of multilayered polymer films, the impact of thermal aging on the mechanical behavior of micro- and nano-layered polymer films has been investigated experimentally. The composition of the polymer films that have been studied is 50 vol% polycarbonate (PC) and 50 vol% poly(methyl methacrylate) (PMMA). The current study focuses on the effect of film and layer thickness and temperature on the mechanical properties of the materials subjected to thermal aging. To study the effect of film and layer thickness, films with the same thickness, but various layer thicknesses, and films with the same layer thickness, but various film thicknesses, were thermally aged at 100 °C in a constant temperature oven for up to six weeks. The results show that as the layer thickness decreases to 31 nm, the film has a higher stiffness and strength, and the trend of the mechanical properties is relatively stable over aging. The ductility of all of the films decreases with aging time. To study the effect of temperature, the films with 4,096 layers (31 nm thick for each layer) were aged at 100 °C, 115 °C and 125 °C for up to four weeks. While the 100 °C aging results in a slight increase of the stiffness and strength of the films, the higher aging temperature caused a decrease of the stiffness and strength of the films. The ductility decreases with the aging time for all of the temperatures. The films become more brittle for higher aging temperatures.

## 1. Introduction

Polymers have a wide range of applications due to their inherent properties, such light-weight, strength, resistance to chemicals, thermal resistance, *etc*. Many processing techniques have been developed to advance the material properties and extend the range of applications. Multilayered polymers have been developed by coextrusion of polymeric systems [1]. The coextrusion technology was first developed by the Dow Chemical Company in the 1970s [2,3,4,5,6]. Multilayered polymers include microlayered and nanolayered polymer depending on the scale of the thickness of layers. Multilayered polymers have been reported to have improved mechanical properties, such as ductility and impact strength, as the layer thickness is reduced [7,8,9]. The microlayered polymer films have been successfully used in industrial applications, such as food packaging and coating [10,11,12]. The 3M Company adopted multilayered polymers for light management in mirrors and the screens of laptop computers. These types of polymer films have some unique properties in their applications. For example, the films can retain the aroma, flavor and freshness of food when used in food packaging. As the coextrusion techniques have advanced in recent years, innovative nanolayered polymer films have been developed, which have more complex hierarchical systems and a truly biomimetic nature [13,14]. There are many potential applications for the nanolayered films, such as gas barrier materials, next-generation flat-panel displays and spherical gradient refractive index (GRIN) lenses [15,16]. To mimic the structures of eyes in nature, polymer lenses made of nanolayered polymer film with GRIN distributions have been developed in recent years. The GRIN crystalline lenses in biological eyes, such as human being’s eyes, typically contain approximately 22,000 layers [17,18]. Layered polymeric optical lenses have been reported to have better optical properties than glass lenses and have been used to replace the traditional glass lens [17,18]. Some researchers have studied the physical and mechanical properties, such as transparency and flexibility, of the novel thin films, although the reports are relatively few [19,20,21,22].

Due to the intrinsic structure of polymers, the mechanical properties of the polymer films can vary widely depending on the material formulation, environmental temperature and time. To facilitate the application of polymers, the deterioration of the material over time, especially at elevated temperature, needs to be studied. We have previously carried out a preliminary investigation to experimentally study the impact of thermal aging on the mechanical behavior of the micro- and nano-layered polymer films [23]. However, in the previous study, all of the films have various layer thicknesses and film thicknesses. This makes it hard to study the effect of layer thickness and film thickness on the change of properties after aging separately. In the current study, the films have either the same film thickness with various layer thicknesses or the same layer thickness with various film thicknesses. This way, the effect of film and layer thickness on the aging of the film can be studied independently. The effect of aging temperature on a specific nanolayered film has also been studied.

The composition of the polymer films under study is 50 vol% polycarbonate (PC) and 50 vol% poly(methyl methacrylate) (PMMA). The layer thickness ranges from 31 nm to 1,984 nm with a 5-μm film thickness and a film thickness of 31.8 μm to 508 μm with a 496-nm layer thickness. These films were thermally aged at 100 °C in a constant temperature oven for up to six weeks. Their mechanical properties, including the modulus of elasticity, tensile strength and ductility, were compared. It has been observed that the thermal aging temperature and aging time have significant effects on the overall character of the stress-strain responses, and layer and film thickness play important roles. The films with 4,096 layers (31 nm thick for each layer) were aged at 100 °C, 115 °C and 125 °C for up to four weeks to study the effect of aging temperature. It has been observed that the film with a 31-nm layer has relatively stable mechanical properties. The microstructural scale level contributes to the material mechanical properties, such as the mechanical stability and durability. The microstructural features start to play a role as the layer thickness reduces to a certain level, which is very important for applications, such as in GRIN lens designs.

## 2. Materials and Tests

**Figure 1:**
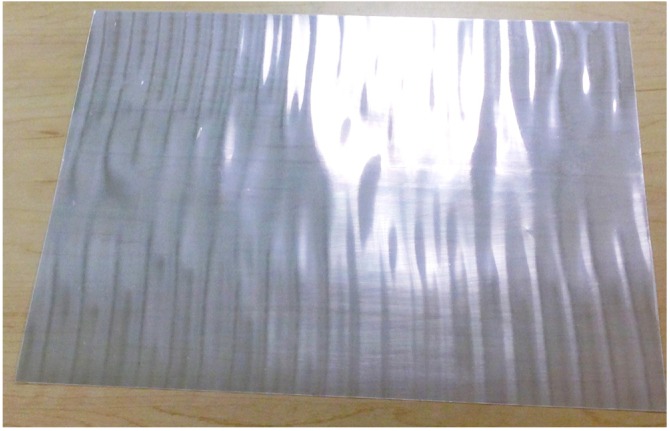
The sample of the multilayered film.

The multi-layered polymer films under study were fabricated with the unique layer-multiplying coextrusion process at Case Western Reserve University. The film sample is shown in Figure 1. The process combines 50 vol% PC and 50 vol% PMMA as perfectly alternating layered systems. The films with various layer thicknesses are shown in Table 1 and films with various film thicknesses in Table 2. The room temperature modulus of elasticity, tensile strength and fracture strain (as a measurement of ductility) of the films were studied with static tension tests using an Instron 4467 instrument in tension mode fitted with film tension grips. The film samples were prepared and tested as directed by ASTM D882. Samples for the tension test were approximately 6 in (152 mm) long and 0.2 in (5 mm) wide. The gage length was approximately 4 in (102 mm). To study the effect of thermal aging, these films were aged 100 °C in a constant temperature oven for up to six weeks. Samples were removed from the oven at aging times of 3 days, 1 week, 2 weeks, 3 weeks, 4 weeks, 5 weeks and 6 weeks (42 days) and tested. The test matrix and sample numbers for each test are listed in Table 3. The test matrix was designed to test about 11 samples for each type of film for aging times up to 42 days. Film samples with 0 days of aging are pristine samples without any aging. Type 4 films were aged to 115 °C and 125 °C. Five samples were removed from the oven at each aging time of 3 days, 1 week, 2 weeks, 3 weeks, 4 weeks, 5 weeks and 6 weeks (42 days) and tested. Note that films with an aging temperature of 125 °C and an aging time of 2 weeks or longer could not be used for the tension test. These films became warped and very brittle after removing from the oven.

**Table 1:** Films with various layer thicknesses.

Type	Number of Layers	Film Thickness (μm)	Layer Thickness (nm)
1	64	127 (5 mil *)	1984
2	256	127 (5 mil)	496
3	1,024	127 (5 mil)	124
4	4,096	127 (5 mil)	31

* mil is a thousandth of an inch.

**Table 2:** Films with various film thicknesses.

Type	Number of Layers	Film Thickness (μm)	Layer Thickness (nm)
5	64	31.75 (1.25 mil)	496
2	256	127 (5 mil)	496
6	1,024	508 (20 mil)	496

**Table 3:** Test matrix showing sample numbers at each aging time for testing for each sample type at 100 °C aging temperature.

Aging Time (day)	Multi-Layer Polymer PC/PMMA Films					
	Type 1	Type 2	Type 3	Type 4	Type 5	Type 6
0	11	13	11	13	11	11
3	8	8	8	9	8	9
7	8	8	8	8	8	9
14	8	8	8	8	8	8
21	8	8	8	8	8	8
28	8	8	8	8	8	10
35	8	8	8	9	8	8
42	8	7	8	8	8	8

The modulus of elasticity was determined from the early linear portion of the stress-strain curves; tensile strength was determined from the maximum stress of the curves; and the fracture strain was the strain when the films finally broke. Film samples in Table 3 were tested, and the data are reported in the next section.

## 3. Results and Discussion

### 3.1. The Effect of the Layer Thickness

**Figure 2:**
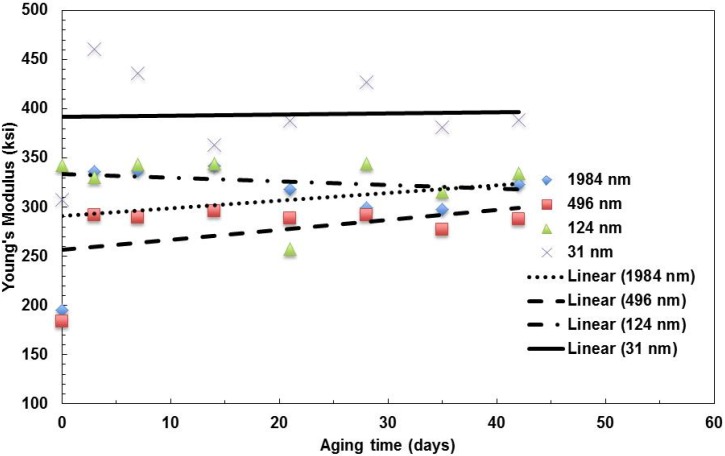
Summary of the change in Young’s modulus with aging time for films with various layer thicknesses aged at 100 °C.

For each test in the test matrix, the results were compiled and the average values were compared. The data for the modulus of elasticity *vs.* aging time at 100 °C are summarized in Figure 2. It is observed that aging has an effect on the modulus of elasticity of films. The significance of this effect changes with the layer thickness. The modulus increases with the aging time to reach about 10%, increases at 42 days of aging for the films with a layer thickness of 1,984 nm and 496-nm films, but decreases for the one with a 124-nm layer thickness. The trend is stable for the films with a 31-nm layer thickness. The modulus value of the 31-nm layer film is the highest among the four types of films. This is because as the layer thickness reduced to 31 nm, the molecular chains of PMMA and PC are more aligned.

**Figure 3:**
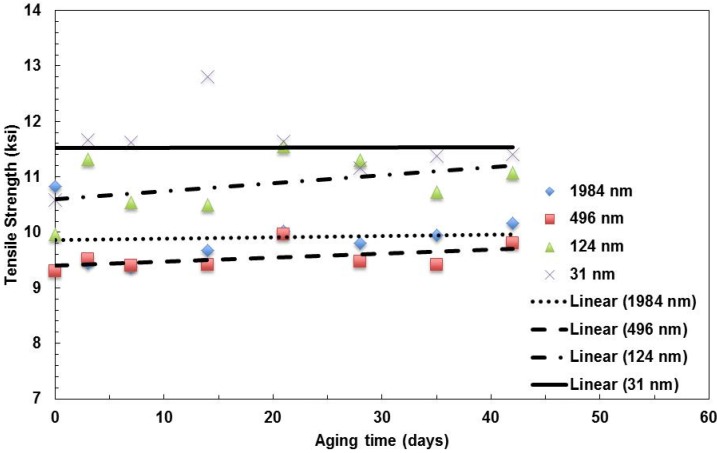
Summary of the change in tensile strength with aging time for films with various layer thicknesses aged at 100 °C.

The data for the tensile strength *vs.* aging time at 100 °C are summarized in Figure 3. The trend of strength does not change with aging time for the film with a 31-nm layer thickness, but it increases with the aging time for the other three types of films. Similar to the modulus of elasticity, the strength of the 31-nm layer film is the highest among the four types of films.

**Figure 4:**
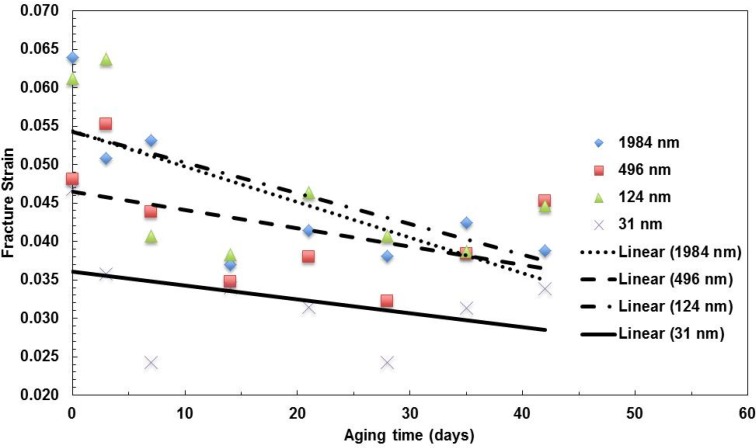
Summary of the change in fracture strain with aging time for films with various layer thicknesses aged at 100 °C.

The data for the fracture strain *vs.* aging time at 100 °C are summarized in Figure 4. The trend of ductility decreases with aging time for all of the films. The ductility was measured by the amount of strain that the material can sustain before it fractures. The thermal aging conditions destroy the covalent bonds in the molecular chains of the polymers. The 31-nm layer film has the lowest ductility among the four types of films. As the film layer reduced to a certain level, the molecular chains of the polymers are aligned and confined in the nanolayer without much ability to be stretched.

### 3.2. The Effect of the Film Thickness

**Figure 5:**
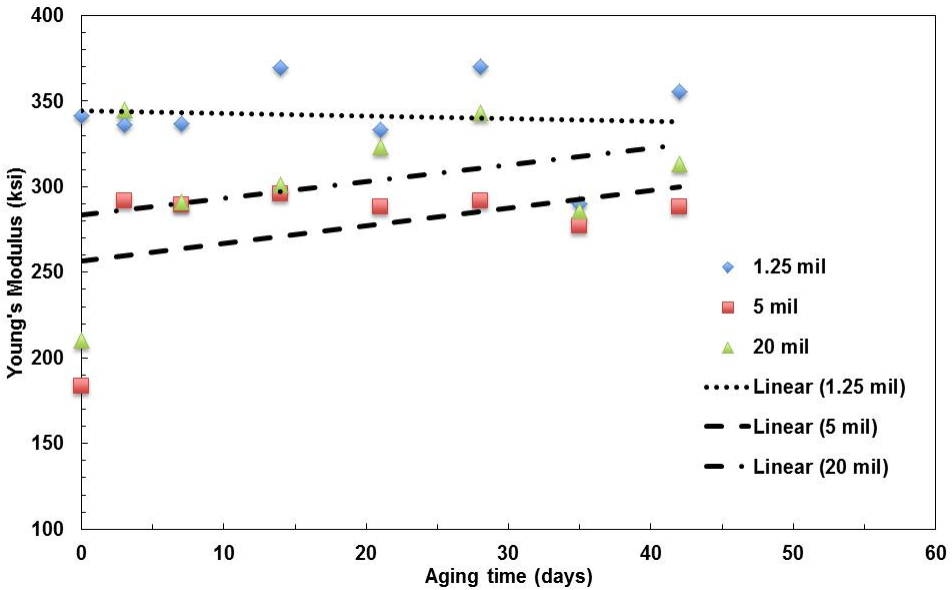
Summary of the change in Young’s modulus with aging time for films with various film thicknesses aged at 100 °C.

The three types of films with a 496-nm film thickness have been tested to investigate the effect of the film thickness. The moduli of elasticity of films with the same layer thickness, but various film thicknesses, are shown in Figure 5. It seems that for the relatively thick films (5 mil and 20 mil thickness films) the pristine film (zero days of aging) have relatively low moduli, which increases with the aging time. The values become relatively stable after aging over three days. For the relatively thin film (1.25 mil), the modulus slightly decreases with aging time.

**Figure 6:**
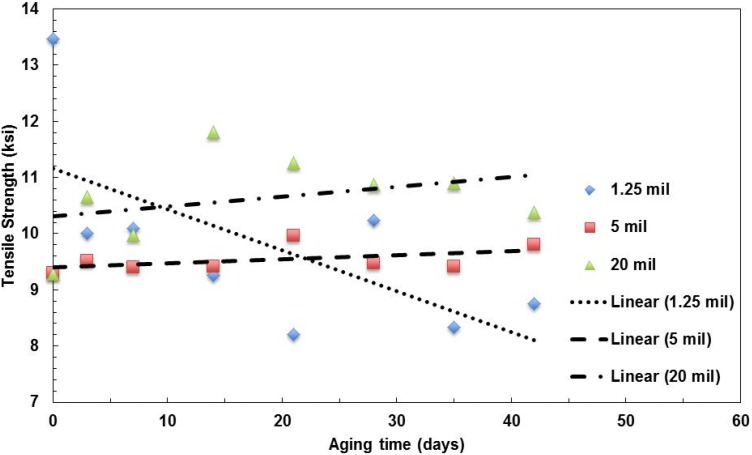
Summary of the change in tensile strength with aging time for films with various film thicknesses aged at 100 °C.

The strength values of the three types of films are shown in Figure 6. The trend of the thin film (1.25 mil thick) is different from the other two types of films. The strength decreases with aging time, but the other two slightly increase with the aging time.

**Figure 7:**
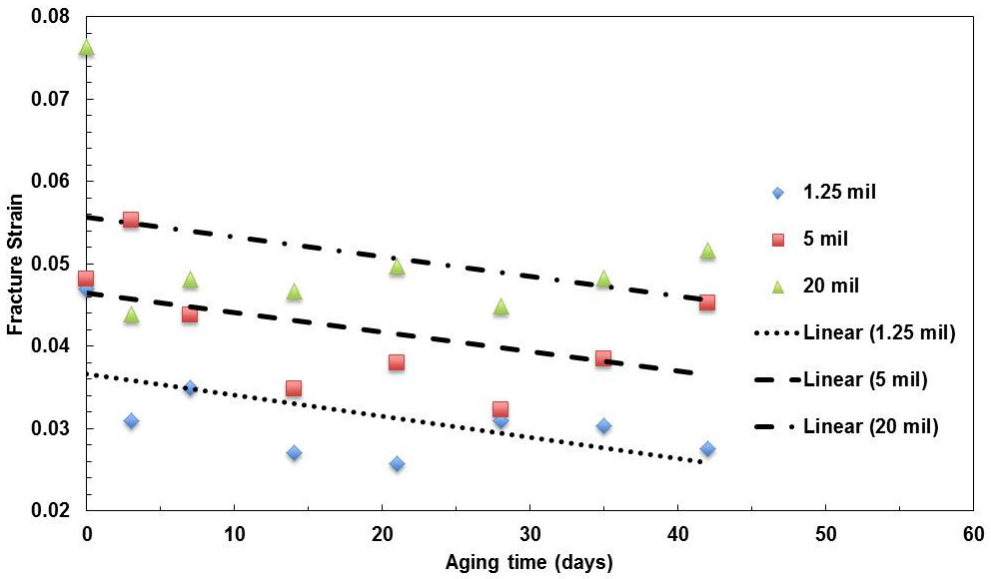
Summary of the change in fracture strain with aging time for films with various film thicknesses aged at 100 °C.

The fracture strains of the films are shown in Figure 7. The ductility of the three films decreasing with aging time shows the degradation of the polymer films.

### 3.3. The Effect of the Aging Temperature

**Figure 8:**
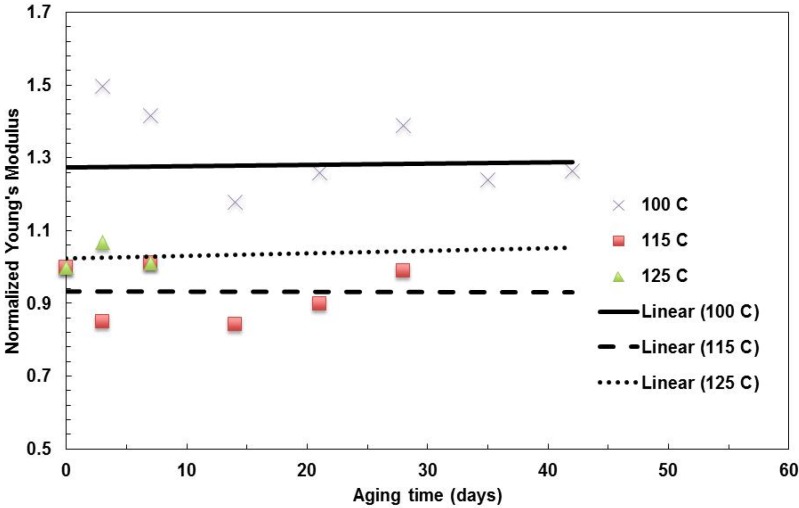
Summary of the change in Young’s modulus with aging time for films with a 31-nm layer thickness aged at various temperatures.

The effect of temperature was studied for the nanolayer film with 4,094 layers of 31-nm thick (Type 4 film in Table 2) at temperatures of 100 °C, 115 °C and 125 °C. While the samples for the aging tests at 100 °C are not from the same batch as the sample for the 115 °C and 125 °C tests, the mechanical properties are not the same for the pristine (original) films. To compare the trend of films from different batches, all of the data were normalized by the pristine material properties. That is, all of the pristine properties are one for zero days without aging. The moduli of elasticity of the aging at three temperatures are shown in Figure 8, from which it can be observed that the trend for the stiffness stays relatively stable over aging time for all three temperatures. The aging at 100 °C has a higher average moduli than the other two.

**Figure 9:**
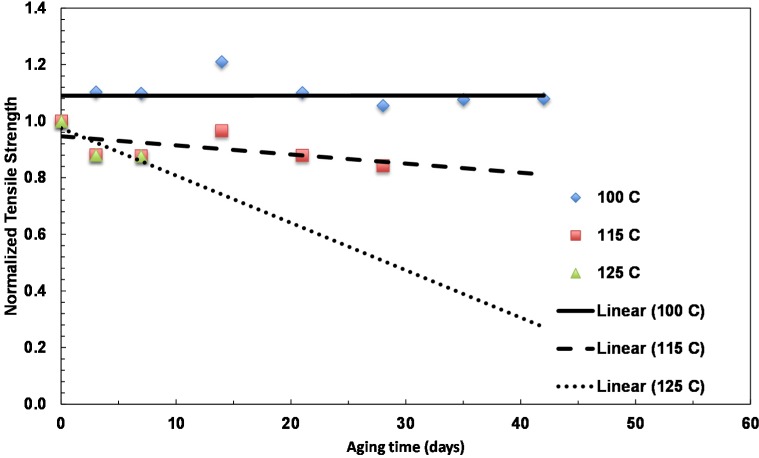
Summary of the change in tensile strength with aging time for films with a 31-nm layer thickness aged at various temperatures.

The tensile strengths of the films are shown in the Figure 9. While the aging resulted in a noticeable decrease for the 125 °C aging, there is no obvious change for the 100 °C aging for the film. The 115 °C aging cause a slight decrease of the property.

**Figure 10:**
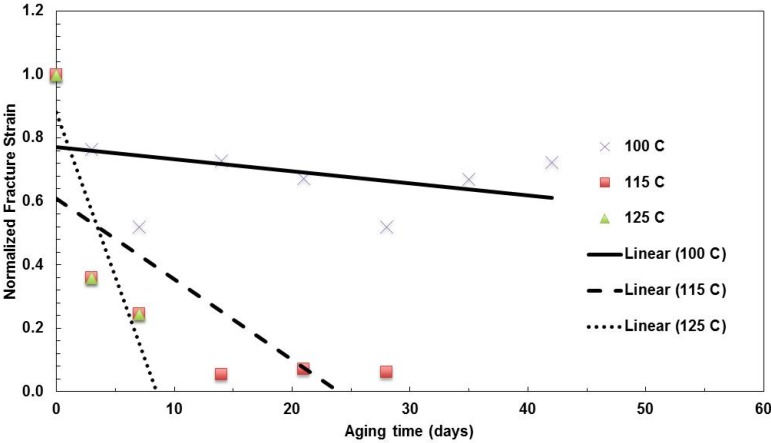
Summary of the change in fracture strain with aging time for films with a 31-nm layer thickness aged at various temperature.

The fracture strains for all three aging temperatures are shown in Figure 10. As all of the films lost ductility during aging, the higher the temperature and the longer the aging time, the more brittle the films become over aging, which indicated the greater degradation of the nanolayer films.

## 4. Conclusions

In this paper, the effects of layer and film thicknesses and thermal aging on the PC/PMMA micro- and nano-layered films have been studied experimentally. It is observed from the results that the material layered structure has a great effect on the mechanical properties and aging. For the films with the same film thickness, but various layer thicknesses, as the layer thickness goes down to 31 nm, the film has higher stiffness and strength than other films with thicker layers. Meanwhile, the stiffness and strength stay stable with the aging time. As the layer becomes very thin, down to the nanoscale, the confinement from the layer boundaries holds the molecules, makes the molecules more tightly packed and, finally, aligned. The aligned molecules have more inter-molecule van der Waals force, which makes them lock tighter to make the material deteriorate more slowly. As the layer thickness stays the same, the stiffness and strength of thinner films decrease with aging time, but for the relatively thicker films, stiffness and strength increase with the aging time. The aging temperature has a great effect on the strength and ductility for the 31-nm nanolayered film. For all of the films, the thermal aging results in a noticeable reduction in the ductility of the films. The higher temperature results in a high loss of the ductility. The findings of this research work demonstrate the breakthrough of the technology to make nanolayered polymer materials not only increase their stiffness and strength, but also increase their stability and durability. While a major limitation of polymer films is the deterioration of mechanical properties in working environments, this finding would be important for the application of the polymer films.

## Figures and Tables

**Figure A1:**
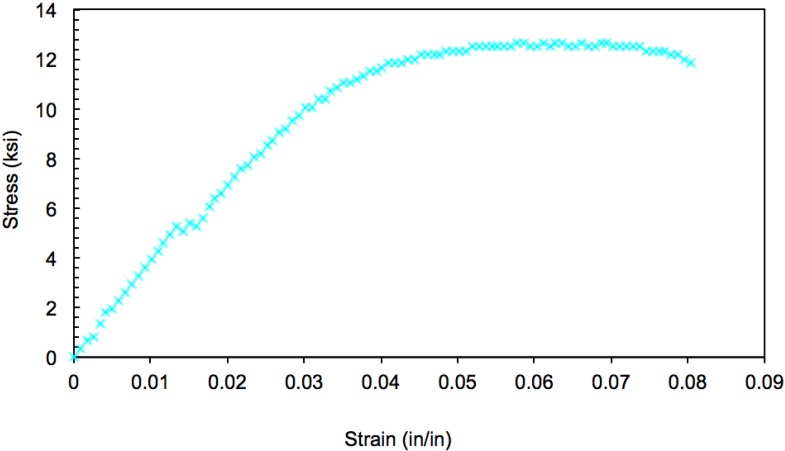
Stress-strain curve.

## Appendix

In this Appendix, testing data for some tests are shown to illustrate the data processing procedures. The data for all of the plots in Figure 2, Figure 3, Figure 4, Figure 5, Figure 6, Figure 7, Figure 8, Figure 9 and Figure 10 are shown in the Table A1, Table A2, Table A3, Table A4, Table A5, Table A6, Table A7, Table A8 and Table A9 for reference.

The static tensile tests at room temperature were done for all of the pristine and thermally-aged PC/PMMA multi-layered films. A tensile curve of a Type 4 film sample (31 nm thick, 4,096 layers) is shown in Figure A1. This curve just serves as an example of all of the tests. The values for the modulus of elasticity, tensile strength and fracture strain of each film are obtained from the tensile curves and compiled.

Table A1 shows the data values for the modulus of elasticity, tensile strength and fracture strain for all types of films at various aging times and temperatures. These are the data for Figure 1, Figure 2, Figure 3, Figure 4, Figure 5, Figure 6, Figure 7, Figure 8 and Figure 9.

**Table A1:** The modulus of elasticity (unit: ksi) *vs.* aging time at a 100 °C aging temperature for film Types 1–4, which have the same film thickness (5 mil), but various layer thicknesses.

Aging Time (days)	0	3	7	14	21	28	35	42
Film Type 1	195	336	337	342	318	300	297	323
Film Type 2	184	292	289	296	289	292	277	288
Film Type 3	343	330	344	344	258	345	316	334
Film Type 4	308	460	436	363	388	427	382	389

**Table A2:** Tensile strength (unit: ksi) *vs.* aging time at a 100 °C aging temperature for film Types 1–4, which have the same film thickness (5 mil), but various layer thicknesses.

Aging Time (days)	0	3	7	14	21	28	35	42
Film Type 1	10.83	9.45	9.37	9.68	10.02	9.81	9.95	10.17
Film Type 2	9.30	9.51	9.41	9.42	9.97	9.47	9.42	9.81
Film Type 3	9.96	11.32	10.53	10.50	11.55	11.31	10.73	11.08
Film Type 4	10.58	11.66	11.62	12.80	11.64	11.16	11.38	11.41

**Table A3:** Fracture strain *vs.* aging time at a 100 °C aging temperature for film Types 1–4, which have the same film thickness (5 mil), but various layer thicknesses.

Aging Time (days)	0	3	7	14	21	28	35	42
Film Type 2	0.064	0.051	0.053	0.037	0.041	0.038	0.042	0.039
Film Type 3	0.048	0.055	0.044	0.035	0.038	0.032	0.038	0.045
Film Type 4	0.061	0.064	0.041	0.038	0.046	0.041	0.039	0.045
Film Type 6	0.047	0.036	0.024	0.034	0.031	0.024	0.031	0.034

**Table A4:** modulus of elasticity (unit: ksi) *vs.* aging time at a 100 °C aging temperature for films with the same layer thickness, but various film thicknesses.

Aging Time (days)	0	3	7	14	21	28	35	42
Film Type 5	341	336	337	370	333	370	290	356
Film Type 2	184	292	289	296	289	292	277	288
Film Type 6	210	345	291	301	324	343	286	314

**Table A5:** Tensile strength (unit: ksi) *vs.* aging time at a 100 °C aging temperature for films with the same layer thickness, but various film thickness.

Aging Time (days)	0	3	7	14	21	28	35	42
Film Type 5	13.47	10.01	10.09	9.26	8.20	10.24	8.33	8.75
Film Type 2	9.30	9.51	9.41	9.42	9.97	9.47	9.42	9.81
Film Type 6	9.28	10.66	9.98	11.81	11.26	10.87	10.90	10.38

**Table A6:** Fracture strain *vs.* aging time at a 100 °C aging temperature for films with the same layer thickness, but various film thicknesses.

Aging Time (days)	0	3	7	14	21	28	35	42
Film Type 5	0.047	0.031	0.035	0.027	0.026	0.031	0.030	0.028
Film Type 2	0.048	0.055	0.044	0.035	0.038	0.032	0.038	0.045
Film Type 6	0.076	0.044	0.048	0.047	0.050	0.045	0.048	0.052

**Table A7:** Modulus of elasticity of the nanolayered film with 4,094 layers of 31 nm thick (Type 4 film in Table 2) aged at temperatures of 100 °C, 115 °C and 125 °C. The data were normalized by the pristine material properties.

Aging Time (days)	0	3	7	14	21	28	35	42
100 °C	1.00	1.50	1.42	1.18	1.26	1.39	1.24	1.26
115 °C	1.00	0.85	1.01	0.84	0.90	0.99		
125 °C	1.00	1.07	1.01					

**Table A8:** Tensile strength of the nanolayered film with 4,094 layers of 31-nm thick (Type 4 film in Table 2) aged at temperatures of 100 °C, 115 °C and 125 °C. The data were normalized by the pristine material properties.

Aging Time (days)	0	3	7	14	21	28	35	42
100 °C	1.00	1.10	1.10	1.21	1.10	1.05	1.08	1.08
115 °C	1.00	0.88	0.88	0.97	0.88	0.84		
125 °C	1.00	0.88	0.88					

**Table A9:** Fracture strain of the nanolayered film with 4,094 layers of 31 nm thick (Type 4 film in Table 2) aged at temperatures of 100 °C, 115 °C and 125 °C. The data were normalized by the pristine material properties.

Aging Time (days)	0	3	7	14	21	28	35	42
100 °C	1	0.76	0.52	0.73	0.67	0.52	0.67	0.72
115 °C	1	0.36	0.24	0.05	0.07	0.06		
125 °C	1	0.36	0.24					

## References

[1] Ponting M., Hiltner A., Baer E. (2010). Polymer nanostructures by forced assembly: Process, structure, and properties. Macromol. Symp..

[2] Schrenk W.J., Chisholm D.S., Cleereman K.J., Alfrey T. (1971). Method of Preparing Multilayer Plastic Articles. U.S. Patent.

[3] Alfrey T., Schrenk W. (1973). Highly Reflective Thermoplastic Bodies for Infrared, Visible or Ultraviolet Light. U.S. Patent.

[4] Schrenk W.J. (1975). Apparatus for Multilayer Coextrusion of Sheet or film. U.S. Patent.

[5] Schrenk W.J., Shastri R.K., Ayres R.F., Gosen D.J. (1992). Interfacial Surface Generator. U.S. Patent.

[6] Ramanathan R., Schrenk W.J., Wheatley J.A. (1993). Coextrusion of Multilayer Articles Using Protective Boundary Layers and Apparatus Therefor. U.S. Patent.

[7] Gregory B., Hiltner A., Baer E., Im J. (1987). Dynamic mechanical behavior of continuous multilayer composites. Polym. Eng. Sci..

[8] Gregory B., Siegmann A., Im J., Hiltner A., Baer E. (1987). Deformation behavior of coextruded multilayer composites with polycarbonate and poly(styrene-acrylonitrile). J. Mater. Sci..

[9] Im J., Baer E., Hiltner A., Baer E., Moet A. (1991). High Performance Polymers.

[10] Cheng W., Gomopoulos N., Fytas G., Gorishnyy T., Walish J., Thomas E.L., Hiltner A., Baer E. (2008). Phonon dispersion and nanomechanical properties of periodic 1D multilayer polymer films. Nanoletters.

[11] Wang H., Keum J.K., Hiltner A., Baer E., Freeman B., Rozanski A., Galeski A. (2009). Confined crystallization of polyethylene oxide in nanolayer assemblies. Science.

[12] Guillorya P., Deschainesa T., Hensona P. (2009). Analysis of multi-layer polymer films. Materialstoday.

[13] Ania F., Baltá-Calleja F.J., Henning S., Khariwala D., Hiltner A., Baer E. (2010). Study of the multilayered nanostructure and thermal stability of PMMA/PS amorphous films. Polymer.

[14] Ania F., Puente O.I., Baltá-Calleja F.J., Roth S., Khariwala D., Hiltner A., Baer E., Roth S.V. (2008). Ultra-small-angle X-ray scattering study of PET/PC nanolayers and comparison to AFM results. Macromol. Chem. Phys..

[15] Baer E., Hiltner A., Shirk J.S. (2006). Multilayer Polymer Gradient Index (GRIN) Lenses. U.S. Patent.

[16] Gupta M., Lin Y., Deans T., Baer E., Hiltner A., Schiraldi D.A. (2010). Structure and gas barrier properties of poly (propylene-graft-maleicanhydride)/phosphate glass composites prepared by microlayer coextrusion. Macromolecules.

[17] Song H., Singer K., Wu Y., Zhou J., Lott J., Andrews J., Hiltner A., Baer E., Weder C., Bunch R. Layered polymeric optical systems using continuous coextrusion. Proceedings of the SPIE7467 Nanophotonics and Macrophotonics for Space Environments III.

[18] Beadie G., Shirk J.S., Rosenberg A., Lane P.A., Fleet E., Kamdar A.R., Jin Y., Ponting M., Kazmierczak T., Yang Y. (2008). Optical properties of a bio-inspired gradient refractive index polymer lens. Opt. Express.

[19] Ebina T., Mizukami F. (2007). Flexible transparent clay films with heat-resistant and high gas-barrier properties. Adv. Mater..

[20] Tetsuka H., Ebina T., Tsunoda T., Nanjo H., Mizukami F. (2007). Highly transparent flexible clay films modified with organic polymer: Structural characterization and intercalation properties. J. Mater. Chem..

[21] Podsiadlo P., Kaushik A.K., Arruda E.M., Waas A.M., Shim B.S., Xu J., Nandivada H., Pumplin B.G., Lahann J., Ramamoorthy A. (2007). Ultrastrong and stiff layered polymer nanocomposites. Science.

[22] Bonderer L.J., Studart A.R., Gauckler L.J. (2008). Bioinspired design and assembly of platelet reinforced polymer films. Science.

[23] Shen H., Gannon N.D. Analysis of the mechanical properties of pristine and thermally aged PC/PMMA micro and nanolayered films. Proceedings of the ASME 2010 International Mechanical Engineering Congress & Exposition IMECE2010.

